# Evaluation of an Emergency Department Sexually Transmitted Infection Empiric Treatment and Linkage-to-care Program

**DOI:** 10.5811/westjem.18583

**Published:** 2025-07-13

**Authors:** Victoria R. Bortner, Emily Holbrook, Heather Henderson, Jason W. Wilson

**Affiliations:** * University of South Florida, College of Public Health, Tampa, Florida; † University of South Florida Morsani College of Medicine, Department of Emergency Medicine, Tampa, Florida

## Abstract

**Introduction:**

Rates of sexually transmitted infections (STI), remain high in Hillsborough County, FL. As the emergency department (ED) is frequently used for STI diagnosis and treatment, a local hospital ED implemented a linkage-to-care program using a callback system to ensure that patients with chlamydia, gonorrhea, and/or syphilis received treatment. Our primary aim in this paper was to evaluate implementation of an ED-based STI treatment program by describing empiric, follow-up, and overall treatment rates in STI-positive patients by disease and sex. A secondary aim was to evaluate reasons for undertreatment during the acute-care encounter.

**Methods:**

We conducted this quality assurance project, including a retrospective chart review of electronic health records from 2019–2022, at an urban ED in Hillsborough County, Florida. During this period, we reviewed all records reflecting positive results for chlamydia, gonorrhea and/or syphilis to determine whether empiric treatment was administered in the ED or the patient required coordination for follow-up care. Patients who received empiric treatment or successful follow-up treatment were classified as treated, while those who did not receive successful follow-up treatment were classified as untreated.

**Results:**

A total of 1,170 patients were diagnosed with an STI at an urban, quaternary-care hospital in the county. Of these, 689 (58.9%) had chlamydia, 324 (27.7%) had gonorrhea, 133 (11.4%) had dual gonorrhea-chlamydia, and 24 (2.1%) had syphilis. Rates of STI empiric, follow-up, and overall treatment were 47.1%, 86.1%, and 92.6%, respectively. Empiric and overall treatment rates were highest for male patients (72.3% male, 33.4% female) and patients presenting with gonorrhea (67.6% gonorrhea, 63.9% chlamydia). Follow-up treatment rates were highest for female patients (87.1%) and patients presenting with gonorrhea (87.6%).

**Conclusion:**

Our findings emphasize both the successes and opportunities for improvement of a linkage-to-care protocol to provide treatment access for patients in the ED who test positive for sexually transmitted infections. Given the significant strain on the public health infrastructure in the United States and on our local Department of Health, ED-based linkage programs fill an important gap in healthcare delivery. Going forward, improving overall treatment rates in females and patients with chlamydia or syphilis is warranted.

## INTRODUCTION

Chlamydia, gonorrhea, and syphilis are three of the most common and curable sexually transmitted infections (STI). In 2018, the STI incidence in Hillsborough County, FL, for chlamydia, gonorrhea, and syphilis were 619.0, 161.6, and 16.8 per 100,000 population, respectively.^1^ Each rate represents an increase from the preceding year (excluding gonorrhea). Of Florida’s 67 counties, Hillsborough ranked ninth highest in these rates.^1^ High-risk groups include females and individuals 20–24 years of age for chlamydia; males and individuals 20–24 for gonorrhea; and males and individuals 25–29 for syphilis.^2^ Between 2008–2010 and 2011–2013, there was a 2% increase in the number of ED visits but a 39% increase in visits by patients with an STI diagnosis.^3^

Screening for STI is routine during ED encounters, and this setting remains effective for reaching high-risk groups. However, results may not be readily available due to long lab turn-around times, delaying treatment and risking loss to follow-up after discharge.^4^–^6^ Therefore, empiric STI treatment is recommended for those experiencing symptoms, those who have engaged in sexual activity with a recently infected partner, and those at high risk of becoming lost to follow-up.^7^ Empiric treatment can reduce transmission and prolonged morbidity.^7^

Previous studies have reported a low sensitivity and specificity for STI diagnosis by emergency physicans and advanced practitioner diagnosis, resulting in both overtreatment and undertreatment.^9^–^11^ Overtreatment is concerning given the concern for antibiotic resistance, while undertreatment perpetuates transmission and disease complications.^2^,^7^,^12^ Specifically, untreated women are susceptible to pelvic inflammatory disease, ectopic pregnancy, infertility, and chronic pelvic pain.^2^

In our ED we treat STIs empirically and use a linkage-to-care program to ensure treatment access for STI-positive patients. The purpose of this quality assurance (QA) study was to assess this ED’s performance in treating identified STIs by evaluating empiric, follow-up, and overall treatment rates in STI-positive patients by disease and sex. A secondary aim was to evaluate reasons for undertreatment. We did not evaluate whether patients were compliant with treatment.

## METHODS

### Study Design

We conducted this QA project, which included a retrospective chart review from the electronic health record (EHR) (Epic Systems Corporation, Verona, WI), between September 2019–October 2022 at a large, urban, academic, Level I trauma center in Hillsborough County, FL. The study design and reporting adhered to the Standards for Quality Improvement Reporting Excellence guidelines^13^ and followed the guidelines of Worster and Bledsoe.^14^ Chart abstractors were trained by the medical director of the ED infectious disease linkage-to-care program. They were familiarized with the US Centers for Disease Control and Prevention (CDC) guidelines for STI treatment and use of the EHR to review inbox messages, physician notes, treatments during the clinical encounter, and prescriptions at discharge. Inclusion and exclusion criteria were clear to the abstractors (participants with positive STI testing in late-results inbox), and the variables were well defined (age, EHR-identified sex, results of STI testing, medications in ED, prescriptions at discharge, diagnosis). They used an electronic data capture form for chart abstraction, and the abstracted results were reviewed by the senior author. Because the study was a QA project and not a research hypothesis, blinding was not necessary. The data were objective with no specific areas of interpretation prone to interobserver variation. If a non-standard antibiotic was used, the abstractors asked the senior author whether the antibiotic met the criteria for appropriate STI treatment. Since there were no interpretive abstracted data susceptible to inter-rater reliability, we did not test for inter-rater reliability. The EHR was used for chart abstraction. The sampling was a predefined time range for analysis. If data were not easily found or recognized in the EHR, the abstractor discussed this with the senior author.

**Population Health Research Capsule**

What do we already know about this issue?

*Lab results for sexually transmitted infections (STI) tests may return post-ED discharge, complicating downstream linkage to care if not treated empirically*.

What was the research question?

*What are current patterns of ED-based STI treatment? Can an ED-based linkage program improve STI treatment rates?*

What was the major finding of the study?

*47.1% received empiric treatment. Gonorrhea led in empiric treatment (67.6%), follow-up (87.6%), and total treatment (96.0%)*.

How does this improve population health?

*There are opportunities to increase ED-based empiric treatment. Post-discharge linkage systems can improve care beyond local Department of Health limitations*.

Screening in the ED for STIs is done for all patients with a suspected STI; in this study we focused on patients ≥13 of age who were positive for the non-HIV STIs chlamydia, gonorrhea and/or syphilis. Minors were included because parental consent for STI examination and treatment is not required in the State of Florida. Persons who screened positive for HIV in addition to non-HIV STIs or were living with HIV were not specifically included or excluded in this study. Our ED has a robust, non-targeted, opt-out, serum-based HIV screening program. The HIV and syphilis test results are managed by a linkage team, but we did not analyze those linkage rates in this review. The linkage navigator was made aware of positive STI testing results through an automated pool in Epic that provides notification of all positive test results. Initial data collection was completed by the linkage navigator through manual chart review. The chart abstractors were not blinded to the study hypothesis.

### Ethical Considerations

The University of South Florida Institutional Review Board deemed the projected to be quality improvement/QA.^14^

### STI Workflow

In the ED, patients underwent STI screening at the discretion of the treating clinical team. A urine polymerase chain reaction test was used for gonorrhea and chlamydia. The reverse screening algorithm using a serum sample for treponemal antibody testing followed by rapid plasma reagin was used for syphilis.^7^ Presence of prior STI was not standardized in approach to STI testing or empiric treatment decision-making. However, clinicians may have used previous STI information when making treatment decisions based on general CDC recommendations and guidelines. We did not interview treating physicians, physician assistants, and nurse practitioners in real time; neither did we conduct qualitative chart review.

Linkage navigators share a pooled Epic inbox. All gonorrhea, chlamydia, HIV, hepatitis C virus (HCV), bacterial vaginosis, and syphilis results are sent to that inbox. Each weekday morning, linkage navigators review the inbox. Our ED uses linkage navigators to follow STI results after patient discharge. They confirm whether empiric treatment was provided in the ED. If no treatment was provided, the linkage navigators provide a prescription to the patient’s pharmacy or arrange linkage for further treatment at the Department of Health (DOH) or return to the ED. The linkage navigators, who are funded as part of Gilead Sciences Frontlines of Communities in the United States (FOCUS) HIV- and HCV-screening program, spend 25% of their time using the EHR to follow STI results and coordinate linkage. They communicate their actions via Epic notes in the EHR. If a physician opted for another treatment course, a response via secure chat or Epic inbox messaging was available (eg, return to ED instead of outpatient prescription). Our linkage navigators are often peers who cross work in our opioid use disorder program or are graduate students in public health or anthropology. Ultimately, these tasks will be integrated into our ED pharmacist and hospital transition-of-care late-results process.

We classified STI-positive patients into the empiric or follow-up group. The empiric group included patients who received appropriate treatment during the encounter based on the clinician’s clinical judgment. The follow-up group included patients who did not receive treatment in the ED, thus requiring downstream linkage to treatment. The linkage navigator contacted follow-up patients to provide STI-positive status and counseling regarding treatment options including visiting the ED, the DOH, or their primary care physician. Patients with chlamydia could have had a prescription sent to their pharmacy. Three call attempts were made to the patient’s primary and secondary contact within 72 hours of the visit. Patients who were not successfully contacted were sent a certified postal letter. The linkage navigator reported all cases to the DOH.

The STI treatment was compliant with the 2015 Sexually Transmitted Diseases Treatment Guidelines 2015 and, later, the updated 2021 guidelines.^7^,^8^ To assess the ED’s performance in treating STIs, patients who received empiric treatment or access to follow-up treatment were classified as “treated”; those who did not receive treatment were classified as “untreated.”

### Analysis

We used descriptive statistics to evaluate demographics and treatment rates. Empiric rates were determined by the number of patients treated empirically within the sample. Follow-up rates were determined by the number of patients treated/untreated within the follow-up group. We determined overall rates by the total number of patients treated within the sample. We performed data analyses using SPSS Statistics v29.0 (IBM Corporation, Armonk, NY)

## RESULTS

### Patient Characteristics

Between 2019–2022, 1,170 patients were STI-positive at this ED. The mean age was 26 (range: 13–70), 758 (64.7%) were female, and 63 were pregnant (8.3%). Of these patients, 689 (58.9%) had chlamydia, 324 (27.7%) had gonorrhea, 133 (11.4%) had dual gonorrhea-chlamydia and; and 24 (2.1%) had syphilis. The most common infection in females was chlamydia (523, 69.0%), and in males gonorrhea (173, 42.0%).

### Main Results

A total of 551 (47.1%) patients received empiric treatment. Gonorrhea patients had the highest empiric (67.6%), follow-up (87.6%) and overall treatment rates (96.0%). Treatment rates by disease are represented in Table 1. By sex, males had higher empiric (72.3%) and overall treatment rates (94.9%), while females had a higher follow-up treatment rate (87.1%). Treatment rates by sex are represented in Table 2. Overall, 1,084 of 1,170 (92.6%) patients were treated; 533 of 619 (86.1%) follow-up patients were treated. However, 86 patients (7.4%) remained untreated. Of those, seven (8.1%) were contacted but not treated; 21 (24.4%) could not be contacted via telephone or certified letter; 41 (47.7%) could not be contacted via telephone only; 11 (12.8%) had incorrect documentation of telephone number(s); two (2.3%) did not have documented contact attempts; three (3.5%) were incarcerated, and one (1.2%) was in residential addiction treatment.

## DISCUSSION

Our results emphasize the need for an ED linkage-to-care process, as empiric treatment was not the treatment modality for all patients. The findings of a relatively low empiric treatment rate, with greater rates in males and for gonorrhea infections, align with what ha been reported in previous studies.^9^,^11^,^15^–^16^, ^17^ This is likely because STI-positive females and patients with chlamydia are more likely to present asymptomatically.^2^,^17^ Alternatively, symptomatic females are at greater risk of being misdiagnosed.^15^ Typically in this ED, gonorrhea/chlamydia and chlamydia test samples are collected via pelvic exam and cervical swab collection. Vaginal self-swabs, urine samples, throat, and rectal samples were not commonly collected. Thus, sample type is unlikely to explain the low rate of empiric chlamydia treatment in women. These low rates may be secondary to lack of clear signs and symptoms on exam compared to those in gonorrhea-positive patients.

Multiple EDs have implemented follow-up infrastructures to link non-empirically treated STI-positive patients to treatment. Some EDs require patients to return to the ED, while others allow patients to receive treatment elsewhere.^6^,^10^,^15^,^18^ However, most studies are limited to female populations and/or patients with gonorrhea and chlamydia only.^6^,^10^–^11^,^15^–^16^,^18^ To our knowledge, our results represent treatment rates of one of the largest ED-based sample sizes of STI-positive patients including syphilis diagnoses, minors and adults, and females and males.

Our ED had high follow-up and overall treatment rates using a linkage protocol. This high treatment rate may be related to our use of secondary contact information and a certified letter after failed call attempts. Additionally, patients were not limited to our ED for treatment. The follow-up protocol was similar to that in one other study that, to our knowledge, had the highest follow-up (93.2%) and overall (96.8%) treatment rates for gonorrhea and chlamydia.^16^ Overall, 7.4% of patients remained untreated and undertreatment was greatest for females and patients with chlamydia or syphilis. To improve ED treatment rates, accurate and rapid point-of-care tests are needed to give physicians and advanced practitioners the ease of verifying test results prior to treatment decisions.^11^,^15^,^18^–^19^ However, these tests are not yet available.^19^ For now, lowering the empiric threshold while enhancing the follow-up protocol may be optimal.^11^,^20^ Lowering the threshold to include those with no STI history may be beneficial, as a previous study found this factor to be associated with undertreatment during follow-up.^16^,^20^

To improve our follow-up protocol, we may consider providing patient activation cards and using a call + text message system as both methods have been associated with improving the rates of successful STI notification and subsequent treatment.^21^–^22^ Patient activation cards provide clear discharge instructions and include the linkage navigator’s contact information.^21^ Or, since most patients remained untreated because of failed call attempts, using a call + text message system may be beneficial.^22^

Our local DOH is ultimately responsible for STI follow-up. However, our DOH, like many, is understaffed. The staff is simply unable to maintain close follow-up and rapid treatment options for STI patients. This scenario can increase patient morbidity and transmission, leaving an opportunity for the ED to plug another hole in healthcare delivery in our local community.

## LIMITATIONS

This study had several limitations. First, we did not analyze clinician encounter notes, which could have provided greater insight into the decision-making process. Qualitative review of the clinical encounter notes may offer further insight into the decision not to empirically treat patients or to deviate from CDC guidelines. Second, untreated patients may have received treatment elsewhere, but it could not be verified. Alternatively, for patients with chlamydia, although a prescription was sent, we were unable to determine whether the prescription was received. However, clinicians commonly send prescriptions with the understanding that patients receive them. We must also consider that there were additional STIs that were undiagnosed in this sample given the reference point for positive test results.

Lastly, our results apply to one urban ED population and are not intended to be generalizable. Clinician-level interviews or feedback at time of encounter may add future insights into the gap in guideline implementation. We did not incorporate that level of analysis in this study. Neither did we consider outpatient treatment completion and ED bounceback rates, which could be analyzed in a future study. Those rates are important when considering prescription barriers, bacterial resistance, or more complicated disease states, such as pelvic inflammatory disease, that may not be treated with outpatient oral antibiotics.

## CONCLUSION

Our findings represent the successes and opportunities to improve the linkage-to-care protocol to provide treatment access to STI-positive patients in this ED. Given challenges of healthcare delivery and the burden on the public health infrastructure in many communities, the potential benefits of coordinated downstream linkage programs in similar ED settings that frequently diagnose STIs should be further explored. Going forward, exploring ways to improve treatment rates in females and patients with chlamydia or syphilis is warranted.

## Figures and Tables

**Table 1:** Treatment rates for sexually transmitted infections by disease.*

Treatment rate	Chlamydia n = 689	Gonorrhea n = 324	Dual Gonorrhea-Chlamydia n = 133	Syphilis n = 24
Empiric treatment group – n (%)	246 (35.7%)	219 (67.6%)	85 (63.9%)	1 (4.2%)
Follow-up group – n (%)				
Treated	382 (86.2%)	92 (87.6%)	40 (83.3%)	19 (82.6%)
Untreated	61 (13.8%)	13 (12.4%)	8 (16.7%)	4 (17.4%)
Overall treatment – n (%)	628 (91.1%)	311 (96.0%)	125 (94.0%)	20 (83.3%)

* Treatment rates represent the number of STI-positive patients within each group who received the specified treatment by disease.

*STI*, sexually transmitted infections.

**Table 2:** Treatment rates for sexually transmitted infections by sex.*

Treatment Rate	Male n = 412	Female n = 758
Empiric treatment group – n (%)	298 (72.3%)	253 (33.4%)
Follow-up group – n (%)		
Treated	93 (81.6%)	440 (87.1%)
Untreated	21 (18.4%)	65 (12.9%)
Overall treatment – n (%)	391 (94.9%)	693 (91.4%)

* Treatment rates represent the number of STI-positive patients within each group who received the specified treatment by sex.

*STI*, sexually transmitted infections.
